# Base Characteristics, Preservation Methods, and Assessment of the Genetic Diversity of Autochthonous Breeds of Cattle, Sheep and Pigs in Serbia: A Review

**DOI:** 10.3390/ani14131894

**Published:** 2024-06-27

**Authors:** Radica Djedovic, Dragan Radojkovic, Dragan Stanojevic, Radomir Savic, Natasha Vukasinovic, Mladen Popovac, Vladan Bogdanovic, Cedomir Radovic, Marija Gogic, Nikolija Gligovic, Petar Stojic, Ivan Mitrovic

**Affiliations:** 1 Department of Animal Science, Faculty of Agriculture, University of Belgrade, Nemanjina 6, 11080 Belgrade, Serbia; radodrag@agrif.bg.ac.rs (D.R.); stanojevic@agrif.bg.ac.rs (D.S.); savic@agrif.bg.ac.rs (R.S.); mlp@agrif.bg.ac.rs (M.P.); vlbogd@agrif.bg.ac.rs (V.B.); nikolija.gligovic@agrif.bg.ac.rs (N.G.); ivanmitrovic98@gmail.com (I.M.); 2 Zoetis Veterinary Medicine Research and Development (VMRD), Kalamazoo, MI 49001, USA; natascha.vukasinovic@zoetis.com; 3 Institute for Animal Husbandry, 11080 Belgrade, Serbia; cedomirradovic.izs@gmail.com (C.R.); gogic.marija@gmail.com (M.G.); 4 Institute for Science Application in Agriculture, Bulevar Despota Stefana 68b, 11000 Belgrade, Serbia; pstojic@sbb.rs

**Keywords:** autochthonous breeds, preservation, cattle, sheep, pigs, Serbia

## Abstract

**Simple Summary:**

This review paper presents previous scientific research on the origin, phenotypic characteristics, population size, as well as the genetic diversity of autochthonous breeds of cattle, sheep, and pigs in Serbia. Autochthonous breeds of domestic animals are considered all breeds that have originated in a certain geographical area and are adapted to the living conditions of that area. The mentioned research is of great significance because animal genetic resources are exceptionally important for all countries and populations around the world due to the fact that they manifest productivity through important agricultural products, demonstrate adaptability, especially to climate change, and can be key contributors to food security for future generations. Therefore, it was extremely important to gather in one review the available knowledge about biodiversity and the possibilities of preserving the most important autochthonous species and breeds not only in Serbia but also in the Western Balkans, all with the aim of considering further directions for their sustainable use and continued existence.

**Abstract:**

Preserving local autochthonous domestic animal populations and the products derived from them is a crucial aspect of managing human utilization of the biosphere. This management approach aims to ensure sustainable benefits for both present and future generations. The diversity of autochthonous domestic animal populations plays a vital role in the functionality and sustainability of the food production system. It encompasses both productive and non-productive aspects, contributing significantly to the overall health, nutrition, and food security of the landscape by providing a wide range of animal-derived food resources. Based on the data contained in the Draft Program of Rural Development, a significant presence of more than 44 autochthonous and local breeds of domestic animals has been noted in Serbia. In order to enable the sustainable preservation of local domestic animals, the competent Ministry of Agriculture of the Republic of Serbia has, through a number of projects, implemented models for the preservation of local breeds on farms (in situ), as well as provided technical assistance to small farms that keep animal collections. It also helps the local population to procure animals, conducts product quality research, and provides opportunities to integrate conservation programs through tourism. Given that molecular characterization is a key factor for the preservation of autochthonous breeds, in the Republic of Serbia, DNA markers are used for identification and to investigate the belonging to a specific breeds or strain. All the mentioned activities led to an immediate increase in the number of animals, which is especially true for the autochthonous breeds of cattle (Busha), sheep (Sjenicka, Svrljiska, and Vlach-vitohorn) and pigs (Mangalitsa, Moravka, and Resavka) that are discussed in this paper. In addition to the significant measures undertaken to preserve animal genetic resources (AnGR), it is necessary to continue to work primarily on ex situ conservation in order to prevent the loss of their gene pools. However, regardless of the evident effort that has been made to preserve autochthonous genetic resources in Serbia, we believe that there is still a lot of room for further improvement. This primarily refers to advanced technologies that have not been applied so far, mostly related to the identification of genomic regions associated with economic traits, resistance to diseases, and adaptability to emerging climate changes. In this way, the production capacity and functional characteristics of autochthonous species and breeds of domestic animals in Serbia will be improved.

## 1. Introduction

By its geographical position, the Republic of Serbia belongs to southeastern Europe, more precisely to the western part of the Balkan Peninsula and part of the Pannonian Plain. The northern part of the Republic of Serbia is plains, while the southern, eastern, and western parts are mainly hills and mountains. Hilly terrain covers 38.5% of Serbia, one-third of which is above 1000 m. Agricultural land covers about 66% of the total area of arable land [1]. In Serbia, the fertile lowlands of Vojvodina and the central parts of the country are dominated by the intensive production of cereals and industrial crops, as well as dairy farms and intensively managed pig farms. In the less fertile, mostly hilly, and mountainous regions of southern, eastern, and western Serbia, livestock production is most prevalent and is partly made up of autochthonous genetic resources (AnGR), i.e., local breeds of cattle, sheep, and pigs. Livestock production in Serbia is therefore extremely connected to natural resources, the traditional way of raising domestic animals, as well as the already existing biodiversity of rural areas.

Based on data contained in the Draft Program of Rural Development [2], a significant presence of more than 44 autochthonous and local breeds of domestic animals has been registered in Serbia (7 breeds of horses, 1 breed of donkey, 8 breeds of cows, 3 breeds of goats, 10 breeds of sheep, 18 breeds of pigs, and several breeds of poultry). 

All the listed breeds have specific phenotypic characteristics and are extremely well adapted to the different climatic and geographical conditions of the areas where they originated and where they are still reared today. Traditional meat and milk products are also closely related to them, enabling the development of agrotourism, which is increasingly growing and significantly contributes to the improvement of the economic status of the local population. In order to achieve the long-term and sustainable preservation of local breeds of domestic animals, it is necessary to combine their efficient and balanced economic exploitation with conservation measures [3]. 

The greatest value of these predominantly primitive breeds is that their genetic makeups produce phenotypes with a solid constitution adapted to unfavorable environmental conditions, vital, and resistant to diseases. The diversity of genotypes of a population represents its potential for survival, whereby the disappearance of autochthonous breeds and the genes they carry would irreversibly reduce the genetic diversity contained in their genomes [4,5]. The loss of any local breed means the loss of both genetic diversity and specific traits, which would be difficult to restore [6]. 

The main reason for the decrease in the number of local breeds is the mass breeding of species and breeds of domestic animals in intensive systems under uniform conditions and the industrial character of production [7]. Most highly productive breeds reared today are genetically homogeneous due to intensive primary selection and inbreeding; these genetically uniform populations are much more sensitive to changes in environmental factors [8,9]. The protection of animal genetic resources is important and necessary for the purpose of preserving natural genetic variability, but it also has scientific, economic, ethical, cultural and historical importance, as well as a certain value in terms of the traditional heritage of each area and its peoples [6,10,11]. 

The determination of affiliation, as well as differences between autochthonous local breeds, was until recently based on phenotype, which was often ineffective, because individual breeds may have similar morphological characteristics. Today, more precise methods are used to identify and establish belonging to a certain breed or strain, such as molecular genetics methods, DNA markers, microsatellites, and SNP markers [6]. 

Compared to commercial breeding programs, such programs for local species and breeds must be adapted in such a way as to primarily increase the size of their populations, as well as to improve their specific characteristics and product quality. It should also not be overlooked that these breeds directly affect production sustainability and the survival of populations in rural areas.

The majority of programs and projects for the conservation of animal genetic resources in Serbia were implemented for local breeds of pigs, cattle, and sheep, and they will be discussed separately in this paper.

## 2. Origin, Size of the Population, and Phenotypic Characteristics of Animal Genetic Resources

### 2.1. Cattle

Busha belongs to the group of short-horned cattle originating from Bos brachiceros europaeus. It is also known as Balkan mountain or Illyrian cattle [12,13,14,15]. It spreads over the territory of almost the entire Balkan Peninsula (Serbia, Croatia, Bosnia and Herzegovina, Montenegro, Albania, Greece, and Macedonia) and is raised as a triple purpose breed (for milk, meat, and work). According to Latinović et al. [4], Busha belongs to the primitive breeds that survived in areas of extensive livestock production, where human influence on cattle breeding was very weak. Busha coat color varies from light gray, yellow, and red to black. A special variant is the tiger Busha, with dense, narrow tiger stripes all over the body. Based on the color and territorial distribution of individual Busha populations, differing mostly in coat color, we can talk about Busha strains. The most important strains represented in Serbia are gray Polimska Busha (southwestern Serbia) and red Metohija Busha (southern Serbia and Kosovo). Busha horns and hooves are always dark to black. 

In the territory of Serbia, the reduction in Busha numbers began in the mid-1920s. In mountainous areas, along with the improvement of rearing conditions, Busha was refined by crossbreeding with more productive breeds (Gray Tyrolean, Montafon, and Pinzgave) in the grading up system. New, more productive populations known as special strains (Gatačka Busha) were also created. In lower areas, where rearing conditions were better, Busha was crossed with the Simmental breed, so that a domestic pied breed in the Simmental type was created by interbreeding over several generations [9].

The existing Busha gene pool can be preserved via in situ and ex situ conservation. In situ conservation started in the early 2000s at several locations with the aim of maintaining the Busha population with an unchanged frequency of genes and genotypes for a longer period of time, with the condition of implementing mandatory mating control in the selected herds without applying inbreeding [16].

Unlike commercial breeds, local cattle breeds, often representing smaller domestic subpopulations, frequently lacked comprehensive record-keeping and consistent selection practices within relevant breeding organizations [17].

Today, the size of the entire Busha population in Serbia is over 3.000 animals of all categories. The effective size of the population (N_e_) has been increasing since 2000 and, in 2023, it was 1316.58 (Table 1).

The phenotypic values of the traits of interest of this autochthonous breed of cattle in the studies carried out so far are presented in Table 2.

The relatively high variability of body mass of males and females of the autochthonous Busha breed can be explained by the different diets and conditions of care and keeping at different localities where tests were performed, as well as the different ages of adult animals when body weights were measured.

### 2.2. Sheep

Today, about 1.7 million sheep of all ages are raised in the Republic of Serbia (Institute of Statistics of the Republic of Serbia, 2023) and over 90% of the sheep are owned by small producers [28]. Petrović et al. [28] state that about 80% of the animals that are raised belong to the autochthonous breed of Pramenka sheep.

After domestication, one of the most important routes by which sheep spread from the primary center of domestication (Anatolia) to Europe was via the Balkan Peninsula and further to the Danube basin and the Mediterranean [29]. For this reason, a large number of sheep genotypes emerged on the Balkan Peninsula, with certain specific genetic and phenotypic characteristics and yet with a greater number of common features. We call the entire group of these genotypes by the common name of Pramenka because of the fleece structure, which is made up of strands.

The Pramenka is an autochthonous breed of sheep reared throughout the Balkan Peninsula and is the most important breed of sheep in the Republic of Serbia. This breed was developed over a long period of time under specific breeding and agroecological conditions. Each of these genotypes has unique genetic characteristics as a result of geographic isolation, random drift, artificial and natural selection, as well as adaptation to the climate, nutrition and diseases prevailing in a certain area. Specific rearing conditions in certain regions led to the appearance of microevolutionary adaptations of sheep in those regions and complete adaptation to such rearing conditions [30]. Pramenka sheep reared in the Balkan Peninsula significantly differ from the sheep breeds reared in the rest of Europe, and these genotypes are related to the domestication process and the Balkans being one of the main routes for the spread of sheep to the rest of the European continent [31].

In the Republic of Serbia 10 strains of Pramenka are reared: Sjenicka, Svrljiska, Krivovirska, Pirotska, Bardoka, Sarplaninska, Karakachanska, Vlach-vitohorn, Lipska, and Balusha sheep [32]. Sjenicka, Svrljiska, Krivovirska, Pirotska, Sarplaninska, and Lipska Pramenka sheep received their names from the region or locality in which they were dominantly reared, while Bardoka, Balusha, and Vlach-vitohorn received their names from some of the phenotypic characteristics they possess. Regarding phenotype, they are characterized by a narrow neck, medium body length, modest musculature, and long and narrow head. Trunk length is slightly greater than the height at the withers, and the chest has a medium length and is deep, narrow, and flat. The ribs are flat and extend obliquely backwards. Sheep are usually hornless, while rams are usually horned. Pramenka strains are divided into strains with fine and coarse wool. Pramenka strains with fine wool (Sjenicka, Svrljiska, etc.) are characterized by dense wool and finer fibers, which is why the yield and quality of wool of these strains is better than of those with coarse wool. On the other hand, Pramenka strains with coarse wool have coarser and axial fibers with long and pointed strands [12]. The aforementioned properties related to resistance to harsh environments influenced the wide distribution and abundance of these genotypes, which were produced under fairly modest conditions and enabled the economic survival of agricultural farms in those areas. 

The majority of Pramenka strains are endangered or at risk of extinction. The Ministry of Agriculture is responsible for the protection of autochthonous breeds of all types of domestic animals. By creating agrarian policy measures, the competent Ministry stimulates the breeding of animals of autochthonous breeds via a system of subsidies. In his research, Stojanović [33] states the number of animals that are bred and controlled for each strain of Pramenka included in the protection and conservation program. Of all Pramenka strains raised in the Republic of Serbia, only the Sjenica and Svrljig strains are not threatened with extinction. The other strains are endangered or at risk of extinction, belonging to Degree 3 of endangerment according to FAO. Table 3 contains basic data on the size of the population, production systems of rearing, types of products, and type of conservation of autochthonous sheep.

The diversity of autochthonous sheep populations a plays a vital role in the functionality and sustainability of the food production system. It encompasses both productive and non-productive aspects, contributing significantly to the overall health, nutrition, and food security of the landscape by providing a wide range of animal-derived food resources.

Given that the mentioned strains of the Pramenka breed are grown primarily for the production of meat and milk, Table 4 presents the published results for the most important traits.

### 2.3. Pigs

Mangalitsa belongs to the group of pig breeds for the production of primitive-type fat. There are conflicting opinions about the origin of the Mangalitsa. Some authors state that this breed is an improved Serbian pig breed, Šumadija, which became more productive due to a transition to improved breeding conditions (from the territory of Serbia to the territory of the Austro-Hungarian–Habsburg Monarchy in the first half of the 19th century), primarily nutrition, housing, and care [43]. Today, there are White, Lasasta and Red strains of Mangalitsa, of which the first two are raised in Serbia.

The Lasasta strain (Swallow-Belly) Mangalitsa is considered to have originated in the area of Srem in the vicinity of the town of Ruma, more precisely in the area of the village of Buđanovci in Srem, and that is why there are local names for this strain—Buđanovci pig or Sremska lasa. Recent research indicates that, based on the DNA structure, different strains of Mangalitsa breeds could be treated as separate breeds [44]. Genetic relationships between the Mangalitsa raised in different geographical locations were studied based on ten microsatellite markers. The estimated genetic distances (Da, Ds, and Fst) were the least between Lasasta and White Mangalitsa, while the Red Mangalitsa showed the greatest genetic distance from the previous two strains. Mangalitsa is a medium-sized breed known for its thick curly bristles, resembling the fleece of sheep, and is thus often called the Woolly Pig, which is especially emphasized during the colder part of the year when finer woolly hair grows between bristles and falls from the body during the warmer part of the year (shedding). The specificity of the exterior of this breed is that piglets are born with “liveries”—stripes that, depending on the strain, disappear when piglets are about 10 days old for the White Mangalitsa and 3–4 months for the Lasasta Mangalitsa.

Lasasta Mangalitsa is a late maturing breed that is resistant and well adapted to extensive rearing and housing conditions. It only requires a simple shelter from rain and snow [43]. 

Mangalitsa is raised in a wider area of the Republic of Serbia, mostly along large watercourses [45]. The in situ conservation program for these breeds in the Republic of Serbia began in the mid-1990s (Special Nature Reserve Zasavica, Mačvanski Prnjavor, Vršac). Based on the Main Breeding Program for Indigenous Breeds of Pigs [46], the conservation program for three indigenous breeds of pigs (Mangalitsa, Moravka, and Resavka) is being actively implemented today. 

Based on presented data and domestic by-laws [47], Mangalitsa had the status of an endangered breed in 2023 based on the total number of female breeding animals (N < 15,000), and of a potentially endangered breed based on the effective population size (Group 3—N_e_ > 200 and ≤1000), which is a significantly more favorable situation compared to 2004, when according to the first criterion, it also had the status of an endangered breed, and according to the second, it had the status of a highly endangered breed (Group 2—No > 50 and ≤200).

Moravka and Resavka are pig breeds with combined production traits created as a result of unsystematic crossing of the Serbian Šumadinka and the British Berkshire breeds [48]. In order to create a herd of pigs for pure breeding and partly to improve production characteristics of the Šumadinka, at the end of the nineteenth and the beginning of the twentieth centuries, Berkshire and Great Yorkshire breed pigs were imported to Serbia, mainly to state farms. As a result of the unplanned and unsystematic crossbreeding of the Šumadinka and the Berkshire breeds, several varieties of pigs were obtained, of which two became established, a completely black one, from which the Moravka was created by mating hybrids obtained with each other, and a much smaller number of multicolored animals, from which, using the same breeding method, the Resavka (“Multicolored Moravka”) was created. This is why Moravka was also known as the “Moravka Black English”.

The Moravka has relatively thin, pigmented black skin and thick but sparse black bristles, which are smooth and straight [49]. Longer and thicker bristles are found on the withers, neck, and upper parts of the body, and thinner and shorter bristles on the lower parts of the body. When removing bristles from the skin with hot water, the pigmented epidermis is removed, leaving the skin completely white [48]. The neck is of medium length and often narrow. The body is quite long and often narrow, and the line of the back is slightly convex or straight. Extremities have a medium length and are thin, delicate, and poorly covered with muscle tissue. The only difference in terms of the exterior between the Moravka and the Resavka is in the color, as the Resavka has uneven areas of black and white–yellow colored bristles and skin.

In terms of breed status, based on the total number of female breeding animals, the Moravka pig breed was considered endangered (N < 15,000) in 2023, while based on the effective population size, it is considered potentially endangered (Group 3—N > 200 and ≤1000). Compared to 2004, when according to the first criterion, the breed had the status of critical, and according to the second, it had the status of critically endangered breed (Group I—N_e_ ≤ 50), the current numbers of the breed are significantly better.

The numbers of the Resavka breed are the lowest compared to the previous two local pig breeds (Table 5). As with the previous two breeds, in the last 20 years, a clear trend of population increase can be observed.

Modern pig production is focused on obtaining meat, while in the past, the production of fat was equally, if not more, important. That is why in the breeding programs for these species, special attention is paid to fattening and carcass and meat quality characteristics. Mangalitsa belongs to the type of pig for fat production, while Moravka and Resavka belong to the type with combined production abilities for meat and fat. That is why they are inferior in terms of meat production compared to modern meat production breeds. On the other hand, numerous studies have shown that the quality of meat produced by local breeds has certain advantages compared to modern breeds, especially when it comes to the production of traditional cured meat products. The basic data on the values of phenotypic characteristics and traits of interest of the autochthonous breed of pigs can be found in Table 6.

**Figure 1:**
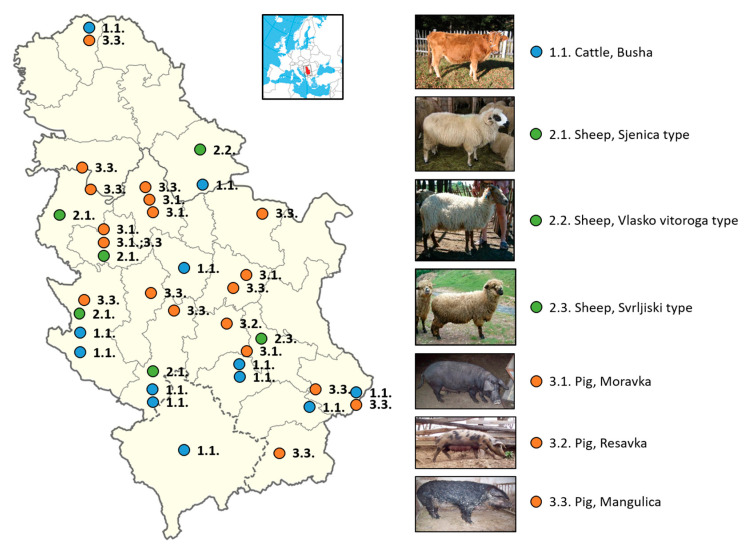
Geographical locations of Serbian local breeds of cattle, sheep, and pigs.

In addition to the most important phenotypic characteristics of indigenous pig breeds determined by a number of authors and given in Table 6, it is necessary to point out that oils and fats are necessary in the human diet; however, health is negatively affected by a too high or too low fat intake. The World Health Organization WHO/FAO [57] reports on the relationship between nutrition and chronic diseases. The human diet should contain 15–30% of energy from fats. Of this, less than 10% should be saturated fatty acids (SFAs) because a higher level increases the contents of cholesterol and triglycerides in the blood. The proportion of polyunsaturated fatty acids (PUFAs) should be 6–10% due to the need for essential fatty acids. According to Petrović et al. [56], the ratio of PUFA n-6/n-3 was higher than favorable and amounted to 18.7 in Mangalitsa and 13.7 in Moravka. Geographical locations of Serbian local breeds of cattle, sheep, and pigs are shown in Figure 1.

## 3. Conservation Strategies and New Biotechnology Methods of Upgrading Animal Genetic Resources

The Food and Agriculture Organization of the United Nations (FAO) has established that over one-third of domestic animals are either extinct or face the threat of extinction and that these domestic animal genetic resources (AnGR) must be conserved, especially in view of the drastic increase in the global human population [58]. The Global Plan of Action for the Conservation of Animal Genetic Resources essentially consists of four strategic areas: characterization, sustainable use, conservation and a national strategy that will enable their continued existence [59]. The National Breeding Program for the preservation of indigenous species and breeds of domestic animals includes two methods of conservation: in situ and ex situ conservation.

### 3.1. In Situ Conservation

By applying in situ conservation, autochthonous breeds in Serbia are preserved by rearing in their original areas, mostly on small private farms. With this primary form of protection, autochthonous breeds successfully express their own ability to live and exhibit their own production characteristics with precise record keeping and strict control of mating in order to prevent inbreeding [22,60,61]. The biggest challenge for conservation of domestic animals in situ is the number of animals that are selected, maintained, and improved under given growing conditions [62,63].

Autochthonous breeds of domestic animals in Serbia are subject to in situ conservation, financed by the Ministry of Agriculture through subsidies for farmers who breed them. The goal of these subsidies is to preserve and restore autochthonous breeds of domestic animals, to enable their long-term adaptation to existing environmental conditions, but also to emerging changes that lead to a series of undesired consequences for the entire ecosystem. In addition to the costs of maintaining animals in their natural environment, it is extremely important to determine the degree of inbreeding in given small populations, namely, to enable the existence of the necessary number of animals of both sexes [64]. Since the effective population size in small herds tends to decrease, FAO recommends a male to female mating ratio of 5:25 for conservation programs. This relationship avoids random changes in gene frequencies and genotypes within populations [9].

### 3.2. Ex Situ Conservation

Ex situ conservation can be carried out in two directions. Ex situ in vivo would mean raising living animals outside their breeding area (in zoos, national parks, etc.). This method of protection has been used for many years under our well-preserved natural conditions. Ex situ in vitro conservation requires a much higher degree of training of personnel and specialized laboratories where genetic material (sperm, eggs, embryos, stem and somatic cells, as well as isolated DNA) would be preserved by cryopreservation [22].

According to Trzcińska et al. [65], today, it is possible to carry out cryogenic preservation or freeze drying (lyophilization) of somatic and stem cells, spermatozoa, oocytes and embryos that are reproduced via assisted reproductive technologies (ART), including artificial insemination, as well as cloning by nuclear transfer of somatic cells and in vitro gamete fertilization. Cryogenic protection of bioresources of somatic and stem cells is also innovative as a condition for the reproduction of domestic animals with the aid of intra- and interspecies cloning by somatic cell nuclear transfer [65,66].

In vitro embryo production and other assisted reproduction techniques (ART) in domestic animals have significantly progressed in recent years. The combination of in vitro procedures with germ cells (germplasm) and the application of genomic selection produces excellent results [64,67]. The number of viable embryos for in vitro production has increased in recent years compared to the number of transferable embryos (multiple ovulation transfer—MOET) produced in vivo [68]. Additionally, tissue cells stored in liquid nitrogen enable partial or complete reconstruction of a breed in the event of its disappearance due to possible natural disasters, and thus enable easier recovery of endangered populations of animal genetic resources. Unfortunately, Serbia is lagging behind in the application of certain methods of assisted reproduction of animals and therefore there are limitations to the full application of ex situ conservation programs because they are extremely expensive [22,69]. In the future, it is necessary to work more on ex situ conservation of animal genetic species and the breeds of domestic animals described in this paper.

### 3.3. Gene Banks

In 2022, Serbia joined the European network of gene banks for animal genetic resources—Eugena [70]. Eugena unites gene banks under the umbrella of the European Regional Focal Point for Animal Genetic Resources with the goal of the ex situ conservation and sustainable use of domestic animals in Europe. It currently covers 14 European countries, with 14 gene banks and 2,070,618 samples (blood, tissue, semen, and embryos). The Institute for Molecular Genetics and Genetic Engineering of the University of Belgrade (IMGGE) currently stores samples of the Busha cow, certain strains of Pramenka sheep, Balkan goats, and Serbian white goats, with the aim of expanding this collection to all autochthonous species and breeds of domestic animals in Serbia.

The goal of creating gene banks is to ensure genetic conservation and future security in the livestock production sector. Samples deposited in gene banks should ensure a wide spectrum of genetic diversity and the genes themselves, primarily for endangered animal breeds. These animal breeds are thought to possess untapped genetic resources containing rare alleles, generally not found in other breeds [71]. Gene banks can also serve as a primary source of material for scientists conducting DNA research. Storing isolated DNA together with samples of spermatozoa, ova, and embryos in the gene bank enables significant scientific research in the field of genetics and genotyping of samples that can be stored for a long period of time [22]. National gene banks are formed based on the recommendations of international institutions and it is necessary to comply with recommended standards. The application of adequate ART methods is standardized, but can be adjusted according to the capacities possessed by individual countries [61].

The future strategy related to gene banks should be directed towards bioinformatic systems that would enable the storage and analysis of genomic estimates of genotyped animals, as well as the monitoring of the interaction between animal genotypes and environmental conditions (GxE). It is considered that gene banks have reduced the costs of preserving populations as live animals (in situ conservation) by more than 90% [60]. Based on the above, it can be considered that the combination of in situ and ex situ conservation methods is the most reliable and effective way of preserving animal genetic resources.

### 3.4. Application of DNA Markers

The success in preserving animal genetic resources would not have been complete if the application of microsatellite molecular markers, which were used to test the origin, individual identification, and genetic diversity of domestic animals, had not started in the last three decades [64,72,73]. These analyses have become very accessible and are successfully used in Serbia. With further development and the advent of genomic analysis, sequencing of whole genomes began with huge advantages such as a short testing time that can be performed at an early age of the animal and at low cost. Single nucleotide polymorphisms (SNPs) have found the greatest application in this field. SNPs can be found within the coding sequence of a gene, but they are also found in non-coding regions. In the coding parts, they can be directly related to the function of the protein, and since these markers are stable when it comes to inheritance, they are very suitable for long-term selection [74]. The obtained data enable scientific conclusions about the general patterns of population structure, ways, and manners, as well as the time of origin of local breeds. Moreover, based on DNA markers, it is possible to judge what the share of natural selection and what the share of artificial selection are in their creation. At the same time, it is possible to identify genomic regions of maximum or minimum divergence between the observed breeds [60]. SNP markers of different densities for the above purposes were used to analyze the autochthonous breeds of pigs and sheep raised in Serbia [75,76,77,78].

The obtained information, especially information on genomic breeding value (gBV), enables expert services, but also farmers themselves, to make decisions about which animals should be used in the nucleus and which in commercial herds to obtain quality traditional products. However, considering that we are dealing with animal genetic resources bred in small populations, where genotization is mainly carried out for commercial breeds, certain errors in the assessment of gBV are possible [79]. The application of genomic selection enables the rapid acquisition of information for traits that are difficult to measure while improving the accuracy of selection and reducing the generation interval [80].

Genomics can also be used to discover potentially valuable rare alleles and haplotypes and their carriers, and help promote and preserve specific genomic regions [79].

### 3.5. Genome Editing and Cloning

Genome editing using methods such as CRISPR/Cas9 allow the deletion, addition or change of alleles at specific locations in the genome of a cell, whereby these changes remain permanent and heritable [81]. An increasing number of scientific studies testify to the possibilities of editing the genome and the genes themselves (GnEd) in order to make specific and precise changes in the genome of an animal and increase productivity or resistance to diseases. The best example in this area is the myostatin gene (*MSTN*), which has become a target for genome editing in domestic animals since changes in this gene have been shown to increase growth and muscle mass in cattle, sheep, goats, and other animals [82,83]. Transgenic technologies and technologies for genome editing are also important for autochthonous breeds, since in a shorter period of time compared to other methods, they can contribute to the improvement of animals by providing new alleles that are not present in highly productive animals but are found in non-domesticated (wild) animal species.

An example is the introduction of an allele for resistance that was introduced from a population of wild common warthogs into domesticated pigs by genome editing [83]. Mueller and Van Eenennaam [84] believe that GnEd should be used to introgress alleles that affect simple so-called Mendelian traits that depend on one or few genes because, in this manner, it is possible to improve them more quickly compared to the application of traditional selection. Quantitative traits of domestic animals that are controlled by a large number of genes, as well as those that are under the significant influence of the environment, are more difficult to improve with these new tools.

**Figure 2:**
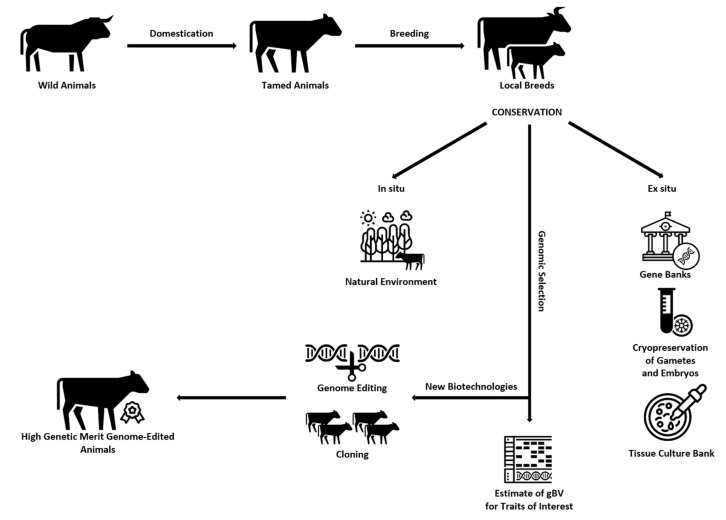
Ways of creating, preserving (in situ and ex situ), and obtaining offspring of high genetic value using different biotechnological methods for improving the traits of interest to breeders.

Graphical representation of the ways of creating, preserving (in situ and ex situ), and obtaining offspring of high genetic value using different biotechnological methods for improving the traits of interest to breeders can be seen in Figure 2.

The process of cloning domestic animals, especially in countries where this method is allowed (North America, South America, and New Zealand), has today become almost routine [84]. The cloning procedure using the somatic cell nuclear transfer (SCNT) technique is performed by removing nuclei from collected ova maturing in vitro. An isolated nucleus from a somatic cell of the animal to be cloned is then introduced into the ovum [67]. The described procedure is repeated on a larger number of ova. Resulting embryos can then be transferred to a surrogate mother or frozen in liquid nitrogen at −196 °C. Theoretically, the number of possible clones that can be thus produced is unlimited [85]. Nicholas and Smith [86] propose three stages through which the cloning of domestic animals and their inclusion in commercial livestock would be carried out. In the first phase, parents would be selected, and their mating would produce offspring that would be potential candidates for cloning. In the second phase, the offspring would be tested in order to evaluate their productive capacities and their breeding value, after which cloning would be conducted. The significant incorporation of cloning into modern breeding programs is limited primarily by the success of this technology and its costs [87]. Unfortunately, most of the described methods of ex situ conservation, as well as more recent biotechnological methods for the improvement of animal genetic resources, such as cloning or genome editing, are not applied in Serbia, but the intention is to adopt and apply some of these methods in the future, such as cryogenic preservation of somatic and stem cells, spermatozoa, oocytes, and embryos of autochthonous species and breeds of domestic animals.

## 4. Application of DNA Analysis for the Identification, Preservation, and Improvement of Animal Genetic Resources in Serbia

### 4.1. Cattle

Methods of molecular genetics and genomic selection, which allow detailed information on the individual structure of the genome to be obtained, have been used in Serbia for a long time to determine the genetic distance between different breeds and strains of domestic animals, to determine the variability of milk protein fractions, as well as to select the most desirable parental pairs to improve traits of interest for breeders.

Using 12 microsatellite markers (TGLA227, BM2113, TGLA53, ETH10, SPS115, TGLA126, TGLA122, INRA23, BM1818, ETH3, ETH225, and BM1824) Stevanov-Pavlović et al. [88] analyzed Busha cattle breeds from the area of Stara Planina, in the vicinity of Dimitrovgrad, taking care that sampled individuals were not related at least two generations back. Using the mentioned markers recommended by ISAG (International Society of Animal Genetics), it was determined that the number of alleles ranges from 6 (for the ETH10 locus) to 16 (for the TGLA122 locus), while the average number of alleles per locus was 9.5. The total number of alleles at all 12 loci analyzed was 114. The obtained results indicate a high and preserved genetic variability of the Busha cattle breed in Serbia.

To assess genetic diversity, Rogić et al. [89] performed a similar analysis on animals of the Busha breed using 21 microsatellite loci. A total of 50 animals included in the research were divided into two groups: Busha from eastern Herzegovina and Busha from western Herzegovina. The average number of alleles per locus was 6.6. The average expected heterozygosity was 0.6885 and 0.6212 in the Eastern and Western populations, respectively. Cluster analysis showed that analyzed populations were grouped into two different classes, which indicates that there is genetic differentiation between the two observed populations.

In Croatia as well as Ivanković et al. [23], the genetic diversity and phylogenetic relationships of three autochthonous breeds of cattle (Busha, Istrian, and Slavonian Srem Podolian cattle) were examined using the mtDNA D-loop region. It revealed 39 polymorphic sites representing 28 different haplotypes. The largest number of haplotypes was observed in the Busha population, and the smallest in the population of Slavonian Srem Podolian cattle. The obtained results indicate a high level of mtDNA diversity in the Busha and Istrian cattle populations. Genetic information based on mtDNA typing is of great importance for the future management of preservation of genetic diversity of indigenous cattle breeds. The obtained results for Busha cattle in Serbia, Bosnia and Herzegovina, and Croatia confirm that the populations inhabiting different geographical regions possess a certain genetic uniqueness that should be maintained and further improved through conservation programs for local cattle breeds.

Given that milk is one of the most important foods in human nutrition, it is extremely important to determine the variability of its protein fractions [90,91]. Cow milk proteins consist of whey protein and casein. Casein makes up about 80% of milk protein and consists of αs1-casein, αs2-casein, β-casein, and κ-casein, while whey protein makes up about 20% of milk protein and consists of α-lactalbumin, β-lactoglobulin (β-lag), and other proteins [92]. κ-CN contains fourteen polymorphic variants, with A and B variants as the most common [93]. The A variant is associated with higher milk yield and a lower protein content, while the B variant is associated with a higher percentage of fat and lower milk yield [94,95].

According to this research, the frequency of κ-CN genotypes AA, AB and BB in Busha cows was 41.7, 50.0, and 8.3%, respectively, while the frequency of alleles A and B, estimated on the basis of genotypic frequencies, was 0.667 and 0.333, respectively. Like in the mentioned research, Marković et al. [95], Brka et al. [96], and Maletic et al. [97] established that the heterozygous κ-casein AB genotype is dominant in the milk of Busha cows, while the research of Ivanković et al. [98] noted the highest frequency of the BB genotype in this breed. Thus, these authors established that the desirable B allelic variant of κ-CN is more prevalent in autochthonous Busha breeds compared to commercial breeds (Holstein Friesian breed), which is an additional reason to protect this breed and further favor it through programs for the production of quality dairy products such as cheeses.

### 4.2. Sheep

For the first time, molecular genetic methods were used to characterize strains in the research conducted by Ćinkulov et al. [72], which aimed to assess the genetic diversity and structure of Pramenka strains reared in the Western Balkans using microsatellite and mitochondrial DNA techniques. The genetic analysis was carried out on a total of 178 animals from 7 Pramenka strains originating from Serbia, Croatia, Montenegro, Macedonia, and Albania. It was found that all observed microsatellite loci were polymorphic, with 185 alleles found in 178 animals. The number of alleles ranged from 6 to 20 for certain loci. The determined average heterozygosity for all loci was 0.781. The genetic differentiation recorded as conventional FST index across Pramenka types indicated that 5.20% of the total genetic variation could be explained by genetic differences among the types. The estimate deviated significantly from zero (*p* < 0.001). The Nei՜s DA genetic distances between the sheep types were calculated and varied from 0.094 (between Svrljig and Recka) to 0.322 (between Istra and Karakachan). All Pramenka animals (178) were divided into six clusters, with the three main clusters encompassing 98% (174) of all animals included in the analysis. The first cluster included all animals of the Svrljig strain and most animals of the Recka, Dubska, Bardoka, and Piva strains. This cluster also had the highest inter-population diversity (0.809), and the average number of alleles was 8.7. As for mitochondrial DNA, 60 haplotypes were established in 64 Pramenka animals. All established haplotypes were classified into two clusters, A and B, comprising 6.3% and 93.7% of the analyzed animals, respectively. The authors concluded that the phylogenetic analysis did not show a clear grouping of mDNA haplotypes according to the Pramenka strain or the region of origin. FST analysis showed that 96.86% of the total number of mDNA control regions were found within Pramenka strains and only 3.14% (*p* = 0.054) outside them. A pairwise comparison of FST showed that the values between Bardoka and the three other strains (Karakachan, Recka, and Svrljig), between Istrian and Karakachan, and between Karakachan and Svrljig Pramenka were significantly different from zero (*p* < 0.05).

Gligović et al. [75] investigated the genetic variability of the Vlach-vitohorn Pramenka sheep strain using genomic tools. Biological samples were collected from a total of 30 animals, 28 ewes and 2 rams. Nasal swabs were used for sample collection, and subsequent genotyping was performed after DNA extraction using the GGP Ovine 50k chip. In terms of data quality control, all animals exhibited a rate exceeding 90%. However, 3572 SNPs were excluded from further analysis due to their minor allele frequency falling below 0.01. Genetic diversity within the studied population was subsequently assessed by examining the distribution of runs of homozygosity (ROHs) across the autosomal genome. A total of 763 ROHs were identified across five ROH length categories, with an average length of 8.09 Mbp. The most extended ROH was found on chromosome 9, while chromosome 3 exhibited the highest proportion of ROH (10.35%). Remarkably, the average genomic inbreeding value (FROH > 16 Mbp = 1.45%) suggested that, despite the relatively small population size (less than 1000 animals), the gene pool does not appear to be significantly impacted by the loss of genetic variability.

### 4.3. Pigs

Researchers from Serbia who participated in the European Union project for research and innovation Horizon 2020; Project No. 634476 (acronym TREASURE) implemented activities related to genomic analyzes of 20 local breeds of pigs (Alentejana and Bísara from Portugal; Iberian and Majorcan Black from Spain; Basque and Gascon from France; Apulo-Calabrese, Casertana, Cinta Senese, Mora Romagnola, Nero Siciliano, and Sarda from Italy; Krškopolje from Slovenia; Black Slavonian and Turopolje from Croatia; Moravka and Lasasta (Swallow-bellied) Mangalitsa from Serbia; Schwäbisch-Hällisches Schwein from Germany; and Lithuanian Indigenous Wattle and Lithuanian White Old Type from Lithuania) from the nine abovementioned countries. 

From these investigations, several studies were created that were primarily related to genomic analyses and assessments of genetic diversity.

In addition to highlighting the importance of genetic diversity for the characterization of the already mentioned 20 local breeds of pigs and a smaller number of Spanish wild boars, Muñoz et al. [76] performed genotyping of 992 DNA samples using high-density SNP chips (GeneSeek Genomic Profiler (GGP) 70 K HD, Illumina Inc., San Diego, CA, USA). The obtained genotyping data were used to estimate genetic diversity, genetic distance, effective population size, as well as the structure of the studied populations.

Results indicate that the local breeds Turopolje, Apulo-Calabrese, Casertana, Mora Romagnola, and Lithuanian autochthonous pig breeds have the lowest genetic diversity, low heterozygosity, as well as a very small effective population size, which requires increased activity on their conservation. Within breeds, observed (HO) and expected (HE) heterozygosity ranged from 0.195 (Turopolje) to 0.363 (Krškopolje) and from 0.187 (Turopolje) to 0.382 (Sarda), respectively. In the mentioned research, the two examined local breeds from Serbia, Lasasta Mangalitsa and Moravka, are genetically very distant. According to the presented neighbor-joining tree, Lasasta Mangalitsa is located in the middle of the western core, while Moravka is almost on the opposite side. Additionally, the two Croatian breeds (Turopolje and Crna Slavonska) are very far from each other. The greatest distance of the Turopolje breeds from all the Balkan pig breeds comes from the fact that it is one of the oldest breeds in Europe because it was domesticated locally in the Middle Ages, while the Crna Slavonska breed was created at the end of the 19th century by crossing the Lasasta Mangalitsa with European noble breeds [99,100]. The effective population size (N_e_) in 50 generations was determined using recombination values for each chromosome. Based on this assessment, wild boars had the highest N_e_ value for 50 generations (521.68), while Mora Romagnola had the lowest N_e_ value (56.63). The Iberian pig N_e_ value indicates the largest current effective population size of the local breeds (89.18), and the Casertana has the lowest value (9.44). The current No for Moravka, in this survey, is 27.25.

Schiavo et al. [77] also evaluated N_e_ for 23 pig breeds using SNEP software (Version 1.11) and obtained similar results. The lowest N_e_ values were for the following breeds: Turopolje, Mora Romagnola, Apulo-Calabrese, and Casertana (N_e_ = 15, 16, 22, and 22, respectively). Similar to Muñoz et al. [76], local breeds with the highest N_e_ were Iberian, Nero Siciliano, Alentejana, Majorcan Black, Sarda, and Bísara (N_e_ = 69, 68, 61, 58, 57, and 55, respectively).

As shown, most local breeds are characterized by a small effective population size, which affects their reduced diversity and leads to a high level of LD (linkage disequilibrium) and a high proportion of SNPs with fixed alleles. The distance between breeds can also be measured by the fixation index (FST). The total FST value for all SNP markers in the study by Muñoz et al. [76] was 0.115, confirming that most of the genetic variation occurred within populations rather than between breeds. These results were also confirmed by Edea et al. [101].

Dadousis et al. [78], in addition to the already mentioned 20 local breeds of pigs that were the subject of research in the TREASURE project, supplemented their work by examining the three most representative commercial breeds (Durok, Landrace, and Large White), as well as data on wild pigs from all 9 participating countries. The goal of the research was to determine the origin of individual breeds through genotyping with high-density chips (70K HD) for genotyping pigs, as well as to assess the traceability of breeds through a discriminant analysis. Although the discriminant analysis confirmed the common origin for Nero Siciliano, Sardo, and Moravka, the research simultaneously indicated an independent origin for the other breeds.

This research also revealed the common origin of some autochthonous and commercial breeds. Durok thus had common ancestors with Cinta Senese, Iberian, and Sarda local pigs; Landrace with Bisara, Moravka, Nero Siciliano, and Sarda; and the Large White with Lithuanian White Old Type pigs. This analysis can simultaneously serve as a history of the domestication of European pigs and indicate crossbreeding between local pig breeds and wild boars. The discriminant analysis confirmed the mixing of Iberian and Italian local breeds with wild boars. The mentioned data were also confirmed by SanCristobal et al. [102].

Using genomic analyses, it is possible to determine the degree of homozygosity through “runs of homozygosity” (ROHs), which is an extremely important parameter when adopting a program to preserve local breeds. ROHs are continuous stretches of DNA without heterozygosity at each polymorphic position. It is believed that the main cause of their formation is inbreeding, and therefore they are a good tool for assessing the level of inbreeding. The existence of ROH segments is explained by the transmission of the same chromosomal segment from parents to offspring that are inherited from a common ancestor. Schiavo et al. [77] investigated the presence of ROHs in a total of 1131 pigs from twenty European local pig breeds and three commercial breeds. Like in previous studies, genotyping was performed using the GGP Porcine HD Genomic Profiler. PLINK software (Version 1.07) was used to identify ROHs. Mora Romagnola and Turopolje breeds had the highest proportions of the genome covered by ROHs (1003 and 955 Mb, respectively), while Nero Siciliano and Sarda breeds had the lowest proportions (207 and 247 Mb, respectively). A comparative analysis of ROH structure among breeds indicated a similar genetic structure of local breeds across Europe. This study contributed to the understanding of the genetic history of the investigated pig breeds and provided information for the management of these pig genetic resources.

The genomic coefficient of inbreeding (FROH), which is defined as the proportion of the autosomal genome covered by ROH segments in the total autosomal genome, in Schiavo et al. [77] showed that Mora Romagnola, Turopolje, Apulo-Calabrese, and Casertana had the highest FROH values. The lowest FROH levels were found in Nero Siciliano (0.085), Sarda (0.101), and Moravka (0.118). This study by Schiavo et al. [77] is extremely significant because it provides insights into the history and genetic origins, as well as the emergence of pig breeds on the European continent. Today, the FROH method is considered superior to other ways of determining inbreeding primarily because it is sensitive to distant inbreeding and takes into account the random nature of inheritance and can be used even if no pedigree data are available. FROH shows better precision in assessing the level of inbreeding, inbreeding depression, and thus better monitoring and the possibility of preserving the population [103].

An analysis of animal genetic material, in addition to revealing genetic distance and inbreeding, is also used to evaluate certain characteristics of animals. In this sense, Núñez et al. [104] analyzed and compared muscle transcriptomes between two autochthonous pig breeds: Lasasta Mangalitsa and Moravka from Serbia. During the test, the differences between the Lasasta Mangalitsa animals that were fed food with added tannins compared to the controls were monitored. A minor effect of dietary supplementation with hydrolysable tannins on muscle transcriptomes in Lasasta Mangalitsa was found. This effect was modest in the number and magnitude of gene expression differences, with an unclear functional interpretation, but pointed to relevant metabolic processes, such as lipid metabolism. Given that this type of research was conducted for the first time in these two domestic breeds of pigs, this research is extremely significant, as it showed a strong effect of breeds on the muscle transcriptome, with relevant genes and metabolic pathways being activated differently in the examined indigenous breeds. At the same time, the main candidate genes, *FLCN*, *PPARGC1B,* and *IL3*, were found to be responsible for the observed phenotypic variations.

Local breeds of pigs, during their creation but also later, were often crossed with wild pigs. In their study, Ribani et al. [105] analyzed melanocortin 1 receptor gene variants (*MC1R*, causing different coat color phenotypes) and group A genes (*NR6A1*, associated with an increased number of vertebrae) in 12 local pig breeds from Italy (Apulo-Calabrese, Casertana, Cinta Senese, Mora Romagnola, Nero Siciliano, and Sarda) and the countries of Southeast Europe (Krškopolje from Slovenia, Black Slavonian and Turopolje from Croatia, Moravka and Swallow-bellied Mangalitsa from Serbia, and East Balkan Swine from Bulgaria), comparing them with wild pigs from the same populations mentioned above. The research showed that none of the indigenous pig breeds or wild boar populations was fixed for one allele at both loci. Domestic and wild alleles of these two genes were present in both domestic and wild populations. Findings on the distribution of *MC1R* alleles show that indigenous pig breeds have a complex and long history that is often associated with inbreeding [106].

As already emphasized, in order to assess the genetic diversity between different autochthonous breeds of cattle, sheep, and pigs reared in Serbia, certain genetic markers were used, the characteristics of which are shown in Table 7.

## 5. Advanced Methods of Analyzing Animal Genomes

In addition to the various microsatellite markers and mitochondrial DNA (mtDNA) applied to assess the genetic diversity of autochthonous breeds of cattle, sheep, and pigs in Serbia and the Western Balkans, in the future, it would be necessary to master and apply new methods, such as resequencing technology of the entire genome and genome-wide association studies (GWAS).

According to Schackwitz and Lipzen [107], whole-genome resequencing is a method to determine differences between an individual and the reference genome of a particular species. Said analysis consists of sequencing an individual’s genome, aligning the generated reads to a common reference population, and detecting variation within the dataset using software tools [108]. Whole-genome resequencing is expected to continue to improve with the goal of using whole-genome sequence data to predict genetic values in domestic animals for traits of interest to producers, and to increase the accuracy of these predictions. When using whole-genome sequencing data, the accuracy of genetic value prediction is increased compared to using high-density SNP arrays. At an equally high density, the inclusion of causative mutations leads to an additional increase in accuracy of 2.5–3.7%. Predictions of genetic value remain accurate even when the data were 10 generations apart [109]. The application of whole-genome resequencing is of extreme importance, especially for animal genetic resources, since this procedure reveals genetic variations and identifies genomic regions associated with economic but also functional traits of domestic animals, such as longevity, resistance to diseases, and adaptation to the environment, especially in a time of significant climate changes and environmental pollution.

As opposed to whole-genome resequencing, genome-wide association studies (GWASs) aim to identify the association between genotypes and phenotypes by testing for differences in the allele frequencies of genetic variants between ancestrally similar but phenotypically different individuals. GWASs typically report blocks of correlated SNPs that show statistically significant associations with the trait of interest, commonly known as genomic risk loci [110]. Within this method, a statistical analysis is performed with the aim of determining the probability that a variant will be associated with the observed trait. The same authors state that the *p*-value indicates the significance of the difference in the frequency of the tested allele between observed cases and controls, namely, the probability that the allele is likely to be associated with the trait of interest. The advantages of implementing GWASs are particularly reflected in small and isolated populations such as populations of indigenous species and breeds of domestic animals. These populations have for prolonged time periods time been suppressed by highly productive breeds, which is why they have limited gene flow. Their advantage is reflected in the fact that their rare functional variants are present in high frequencies [111].

Due to a high coefficient of kinship, which is most commonly present in small and isolated populations of indigenous species and breeds of domestic animals, high genetic homogeneity is observed due to possible losses of alleles passing through so-called genetic bottlenecks, which can increase the power of burden tests by reducing the number of neutral variants [112]. Lost gene variants are very difficult to replicate; therefore, it is extremely important to preserve the diversity of already existing animal genetic resources that are adapted and characteristic for certain countries and regions, as is the case with the described autochthonous breeds of cattle, sheep, and pigs in Serbia and the Western Balkans. 

In addition, it is expected that the integration of whole-genome resequencing data with existing microsatellite markers and mtDNA information will improve the resolution and scope of genetic analyses, which will also improve the practical value of these studies. As can be seen from above facts, there is no doubt that there is great potential hidden in the methods of whole-genome resequencing and association studies at the genome level, and we will strive to make them the subject of our future research. We will also try to include the mentioned methods in future strategies for the conservation and cultivation of animal genetic resources in Serbia.

## 6. Conclusions

There are many unique autochthonous breeds of livestock in the Republic of Serbia, each of which contributes to the genetic diversity and richness of the livestock populations. However, some of them are endangered or on the verge of extinction. The program for the preservation and protection of local breeds of domestic animals in the Republic of Serbia was started in the 1980s, but unfortunately, until the year 2000, the state conservation systems for animal genetic resources (AnGR) applied were economically and technically unsustainable, so that many breeds and strains of domestic animals from the territory of Serbia have almost disappeared. In order to prevent further “genetic erosion” of local breeds, which would lead to an irreversible loss of diversity among autochthonous animal populations in the Republic of Serbia, a number of strategies were implemented, but it is necessary to continue working on the implementation of effective breeding programs for all species of endangered local breeds.

At present, we can directly assess genetic diversity by analyzing DNA, which offers accurate insights into the genetic variations within and between breeds, populations, and individuals. These tools enable the identification of populations or individual animals with unique traits, allowing for targeted protection efforts. However, the measures taken are not enough and therefore we have a serious task ahead of us to apply the new biotechnology methods of upgrading animal genetic resources described here. We also need to integrate existing data on AnGR obtained from studies using microsatellite markers and mtDNA with data such as genome resequencing technology, given that there is great potential with this research, which we hope will be the subject of our future research endeavors. 

At both national and international levels, significant efforts are being made to preserve all food sources for human consumption and agriculture. When it comes to local resources, preserving native breeds is not solely about their uniqueness; it is also about their role in enhancing regional diversity and serving as an indispensable aspect of a nation’s cultural heritage. By prioritizing the conservation of local autochthonous domestic animal populations and their genetic resources, we can safeguard their valuable traits, promote biodiversity, and preserve the cultural and environmental heritage of Serbia, the Western Balkans, as well as worldwide communities.

## Figures and Tables

**Table 1:** Basic data on the size of the population, production systems of rearing, types of products, and type of conservation of autochthonous cattle of the Busha breed.

Local Breed	Busha		References
	Year		
	2004	2023	
Population maximum	1000	3000	[15]
Breeding males	10	403	
Breeding females	90	1796	
N_e_ *	36.00	1316.58	
Animal products	Milk, meat		[4,18,19,20]
Product certification	National certificate with designation of geographical origin for Stara Planina Kashkaval cheese		[21]
Production system	Extensive, semi-intensive		[12]
Type of conservation	In situ and ex situ (gene bank)		[22]

* N_e_—effective population size.

**Table 2:** Values of phenotypic characteristics and traits of interest of the autochthonous breed of cattle—Busha.

Traits	Values		References
	Males	Females	
Height at the withers (cm)	≥120	105–115	[12]
	120	90	[23]
Body mass (kg)	245–301	150	[16]
	350–400	135–264	[24]
	330–350	200–250	[25]
	300–350	250	[26]
Weight of calves at birth (kg)	18–20	14–16	[26]
Daily gain (kg)	0.2–0.6		[20]
Sexual maturity (months)	18	20–25	[12]
Age at first calving (months)	35		[16]
Milk yield in lactation (kg)	800–1500		[26]
Fat content (%)	4–6		[4]
	3.62–4.04		[20]
Protein content (%)	3.72		[27]
Dry matter content (%)	13.47		[27]

**Table 3:** Basic data on the size of the population, production systems of rearing, types of products, and type of conservation of autochthonous sheep.

Local Breed-Pramenka	Sjenica Pramenka Strain		Svrljig Pramenka Strain		Vlach-Vitohorn Pramenka Strain		References
Year	2004	2023	2004	2023	2004	2023	[15]
Population maximum	50,000	300,000	50,000	35,000	/	2000	
Breeding males	0	6559	0	1193	/	42	
Breeding females	0	217,855	0	31,666	/	1096	
N_e_ *	0.00	25,469.19	0.00	4598.74	/	161.80	
Animal products	Meat, milk, wool						[34,35]
Product certification	National certificate with designation of geographical origin for Sjenica Sheep Cheese, Sjenica lamb, and Sjenica litter		National certificates with designation of geographical origin for Svrljig Kashkaval cheese and Svrljig Belmuz Kaymak		/		[36,37,38,39,40]
Production system	Extensive, semi-intensive		Extensive		Extensive		[34,35]
Type of conservation	In situ and ex situ (gene bank)		In situ		In situ		[22]

* N_e_—effective population size.

**Table 4:** Values of phenotypic characteristics and traits of interest for the autochthonous strain sheep breed—Prameka.

Local Breed/Traits	Sjenica Pramenka Strain		References	Svrljig Pramenka Strain		References	Vlach-Vitohorn Pramenka Strain		References
	Males	Females		Males	Females		Males	Females	
Body mass (kg)	100–130	75–100	[34]	65	50	[35]	42	54	[41]
Lamb weight at 30 days (kg)	30	26	[34]	11	13	[34]	10–12		[35]
Sexual maturity- females (months)	7–10		[35]	12		[35]	18		[35]
Lambing index	1.3–1.8		[35]	1.9		[42]	1.2		[41]
Milk yield in lactation (kg)	130–200		[34]	64		[42]	80–110		[41]

**Table 5:** Basic data on the size of the population, production systems of rearing, types of products, and type of conservation of autochthonous breeds of pigs.

Local Breed	Mangalitsa		Moravka		Resavka		References
	Year						
	2004	2023	2004	2023	2004	2023	
Population maximum	1000	4000	1000	5500	100	500	[15]
Breeding males	20	111	5	237	2	20	
Breeding females	200	2349	30	3824	8	204	
N_e_ *	72.73	423.97	17.14	892.67	8.40	72.86	
Animal products	Meat, fat						[50]
Product certification	/		/		/		
Production system	Extensive		Extensive		Extensive		[51]
Type of conservation	In situ		In situ		In situ		[51]

* N_e_—effective population size.

**Table 6:** Values of phenotypic characteristics and traits of interest of the autochthonous breeds of pigs.

Local Breed/Traits	Mangalitsa		References	Moravka		References	Resavka		References
	Males	Females		Males	Females		Males	Females	
Body mass (kg)	190	165	[43]	72–152	70–160	[48]	98	94	[48]
Number of piglets per litter	5		[45]	7.31		[52]	7.96		[52]
	/		/	6		[53]	8		[53]
Sexual maturity—females (months)	8–12		[45]	5–6		[53]	/		/
Daily gain (kg)	0.480		[54]	0.545		[54]	/		/
	0.307		[55]	0.316		[55]			
Saturated fatty acids, SFAs, %	39.45		[56]	41.64		[56]	/		/
Monounsaturated fatty acids, MUFAs, %	56.41		[56]	53.78		[56]	/		/
Proportion of polyunsaturated fatty acids, PUFAs, %	4.10		[56]	4.54		[56]	/		/

**Table 7:** Number of microsatellite markers and density of SNPs used in the assessment of genetic diversity of autochthonous breeds of cattle, sheep, and pigs reared in Serbia.

Aautochthonous Breeds Cattle	Markers	Marker Density	References
Busha	Microsatellite	12 Microsatellite loci	[88]
Aautochthonous breeds sheep			
Sjenicka	Microsatellite	15 microsatellite loci	[72]
Vlach-vitohorn	SNP Markers	The Illumina Ovine 50K chip	[77]
Aautochthonous breeds pigs			
Mangalitsa	SNP Markers	The Illumina Pig 70K HD chip	[76]
Moravka	SNP Markers	The Illumina Pig 70K HD chip	[76,78]

## Data Availability

No new data were created or analyzed in this study. Data sharing is not applicable to this article.
